# Account of an Elastic Trochar, Constructed on a New Principle, for Tapping the Hydrocele or Watery Rupture: By Which the Operation May Be Performed at Any Period of the Disease, and with Less Pain Than with the Common Trochar. With a Few Words in Favour of a Larger Trochar, on a Similar Construction for Tapping the Abdomen

**Published:** 1781-06

**Authors:** 


IV. *Account of an elastic Trochar, constructed on a new principle, for tapping the hydrocele or watery rupture: by which the operation may be performed at any period of the disease, and with less pain than with the common trochar. With a few words in favour of a larger trochar, on a similar construction for tapping the abdomen. By* John Andree, *Surgeon to the Magdalen Hospital, and the Finsbury Dispensary.* 8vo. Davis, *London*, 1781. 41 pages with a copper-plate, 1s.

THE superiority of modern surgery over that of the ancients is in a great measure owing to the various improvements that have been made in the structure of surgical instruments. In the pamphlet before us an account is given of an improvement of the trochar; an instrument, as the author observes, that is more frequently used, than almost any other in surgery, excepting the lancet and scalpel.

It

It has been objected to the common trochar that it not only occasions considerable pain, but likewise that it cannot be used in the early stage of the hydrocele without danger of wounding the testicle. Both these inconveniences, the author assures us, are remedied by his elastic trochar, of which he gives the following description:

"This instrument consists of two parts; the "one is called the stilet, or perforator, the other "the canula. The whole of the stilet, excepting its point, is contained within the canula, "which is flat, but somewhat convex on each "surface, and has two sharp edges.

"The canula (a tube) is formed of two pieces "of well-tempered elastic steel, which are so "accurately fitted together at their edges, as "to form a complete canula, and closely embrace the body of the stilet. When the instrument has been passed into the part affected, "on withdrawing the stilet with the smallest degree of force, the canula opens just wide "enough to allow of its exit, and afterwards, "by its own elasticity, closes immediately; being then a complete and perfect canula, open "at each end."

The author remarks that this new trochar may be used with safety, as soon as there is a depth of water equal to the cutting part of the inftrument, and a very fmall portion of the canula; which depth three or four ounces will afford. He himfelf lately tapped a hydrocele, the contents of which were only fix ounces, although by many authors, the quantity of water to be collected is fixed at a pint.

That the ufe of this new inftrument will be attended with lefs pain than the common trochar, is a point, he thinks, not to be queftioned, when it is confidered that the wound made with the former is, as he has proved by experiments, and by the moft exact meafurement, lefs by about one third than that made by the common inftrument, and that its canula enters without the leaft degree of force. The principal advantage afcribed by Mr. Andree to this laft circumftance is, that the ufe of his trochar will be perfectly fafe in an early ftate of the difeafe, as it entirely fecures the tefticle from the danger of being wounded by the ftilet; of which there is confeffedly fome degree of hazard by the common trochar, in thofe cafes at leaft in which the vaginal coat is become fo very tough and thick, as not to be penetrated without much force in ufing that inftrument, which is then apt to plunge deeper into the part than was intended. This, he imagines, is the circumſtance alluded to by the late Mr. Sharpe, when he recommends, in ſuch caſes, the puncture by lancet, in preference to that by the trochar.

Mr. Andree is perſuaded that an inſtrument on the ſame conſtruction, of a larger ſize, will alſo be found preferable to the inſtrument at preſent uſed in performing the radical cure for the hydrocele by ſeton; both becauſe it would paſs more eaſily, and becauſe it might be uſed at an early period of the diſeaſe, which the other cannot, from its ſize being ſuch as to require a conſiderable degree of reſiſtance to admit of its uſe with ſafety.

He obſerves likewiſe that a ſimilar trochar, of a larger ſize, may be uſed for the aſcites, and will be found to have the ſame advantages over the common one, viz. that it will enter with more eaſe to the operator, and leſs pain to the patient; and that it may be uſed with ſafety in an early ſtate of the diſeaſe.

Early tapping in the abdominal dropſy, though recommended by ſome of the beſt phyſicians, and on the moſt rational principles, has not been hitherto conſidered as a ſafe operation; a conſiderable quantity of water being judged neceſſary to yield ſufficient reſiſtance to the inſtrument, to keep keep it from wounding the viſcera. This exception however, our author is of opinion, may certainly be obviated by the uſe of this trochar, ſince, as it may be introduced without force, a very ſmall quantity of water will ſuffice to keep its point from doing miſchief.

It is certain that under certain circumſtances early tapping may become a moſt deſirable object, in order to relieve the patient from the pain occaſioned by the water, before it is accumulated in ſufficient quantity to render the uſe of the common trochar ſafe, or warrantable. The truth of this obſervation is ſufficiently illuſtrated by the following curious caſe related by the author.—A patient was afflicted with a pain at his ſtomach, which had been gradually increaſing for two years. On the 16th of September, the day Mr. Andree firſt ſaw him, he was troubled with conſtant ſickneſs, and inclination to vomit after taking the ſmalleſt quantity of animal food. His belly was much and ſuddenly increaſed in ſize. A degree of undulation was perceptible by the touch. The quantity of fluid, however, was not ſufficient to warrant the uſe of the common trochar; but the patient moſt earneſtly deſired to be tapped, as the great and hourly increaſe of pain from the accumulating water, rendered life otherotherwife infupportable. In compliance with his requeft, and with the confent of the other medical gentlemen who were confulted, our author ventured to tap him immediately with a common large trochar (not having then invented his new inftrument) after firft making a puncture with an impofthume lancet. This operation was repeated three feveral times. He filled remarkably faft; for he was tapped a fecond time on the 4th of October, and again on the 25th. On infpection of the parts after death it was difcovered that this dropfy was occafioned by a fchirrus of that part of the omentum which is attached to the ftomach. The water had been firft collected between the two lamina of the omentum, which it had burft through, and was diffufed into the cavity of the abdomen. This circumftance happened a few days before the firft operation; and the patient was enabled to fpeak of it, with certainty, from having felt it fo very plainly.

